# Identification of Somatic Mutations From Bulk and Single-Cell Sequencing Data

**DOI:** 10.3389/fragi.2021.800380

**Published:** 2022-01-03

**Authors:** August Yue Huang, Eunjung Alice Lee

**Affiliations:** Division of Genetics and Genomics, Manton Center for Orphan Diseases, Boston Children's Hospital, Boston, MA, United States, Department of Pediatrics, Harvard Medical School, Boston, MA, United States

**Keywords:** somatic mutation, bulk sequencing, single-cell sequencing, bioinformatic tool, single-nucleotide variant

## Abstract

Somatic mutations are DNA variants that occur after the fertilization of zygotes and accumulate during the developmental and aging processes in the human lifespan. Somatic mutations have long been known to cause cancer, and more recently have been implicated in a variety of non-cancer diseases. The patterns of somatic mutations, or mutational signatures, also shed light on the underlying mechanisms of the mutational process. Advances in next-generation sequencing over the decades have enabled genome-wide profiling of DNA variants in a high-throughput manner; however, unlike germline mutations, somatic mutations are carried only by a subset of the cell population. Thus, sensitive bioinformatic methods are required to distinguish mutant alleles from sequencing and base calling errors in bulk tissue samples. An alternative way to study somatic mutations, especially those present in an extremely small number of cells or even in a single cell, is to sequence single-cell genomes after whole-genome amplification (WGA); however, it is critical and technically challenging to exclude numerous technical artifacts arising during error-prone and uneven genome amplification in current WGA methods. To address these challenges, multiple bioinformatic tools have been developed. In this review, we summarize the latest progress in methods for identification of somatic mutations and the challenges that remain to be addressed in the future.

## Introduction

The human body consists of more than 10^13^ cells developed from a single fertilized zygote and experiences about 10^16^ cell divisions throughout its lifespan (Sender et al., 2016). Previously, all the cells from a single individual were thought to carry an identical genome, but this has been proven wrong due to the widespread occurrence of somatic mutations even in healthy individuals (Evrony et al., 2012; Lupski, 2013; Huang et al., 2014). Somatic mutations occur postzygotically as a result of errors in DNA replication and exposure to exogenous and endogenous mutagenic factors (Vijg and Dong, 2020). Once fixed in the genome, somatic mutations can be inherited from parental cells to daughter cells through cell division; when somatic mutations occasionally affect lines of germ cells, the mutations may be transmitted to offspring (Ye et al., 2018). The scale of somatic mutation varies from single-nucleotide variant and short indel to structural variation and chromosomal anomaly, and the somatic single-nucleotide variant (sSNV) is the most common mutation type in the human genome (De, 2011).

Somatic mutations have increasingly been implicated in various diseases. Somatic mutations in oncogenes and tumor-suppressor genes are the major cause of cancer (Watson et al., 2013). Accumulation of somatic mutations in cancer driver genes has also been reported in precancerous and apparently normal samples of blood and epithelial tissues, and is associated with increased cancer risks (Kakiuchi and Ogawa, 2021). In addition to cancer, somatic mutations have been found to play a critical role in an increasing list of non-cancer overgrowth diseases, such as Proteus syndrome (Lindhurst et al., 2011), arteriovenous malformation (Couto et al., 2017), and brain malformation (Jamuar et al., 2014). As a previously overlooked genetic factor, somatic mutation has been implicated in more and more non-Mendelian, complex diseases including autism (Dou et al., 2017; Lim et al., 2017), schizophrenia (Fullard et al., 2019), and congenital heart disease (Hsieh et al., 2020). Using single-cell sequencing, an increased genome-wide burden of somatic mutation in neurons was found to be associated with aging and neurodegenerative conditions (Lodato et al., 2018).

Different mutational processes generate distinct profiles of mutational genomic contexts, termed “mutational signatures,” and the landscape of somatic mutations observed in tissue samples or single cells often reflects the combined impact of multiple mutational processes (Helleday et al., 2014). The large collection of somatic mutations from cancer samples has enabled the decomposition of mutational profiles from different cancer types into mutational signatures. By using non-negative matrix factorization (Lee and Seung, 1999), Alexandrov *et al.* analyzed the tri-nucleotide sSNV profiles across 30 cancer types and successfully identified 27 mutational signatures (Alexandrov et al., 2013). The catalogue of mutational signatures has then been extended by incorporating more cancer data and other mutation types including short indels and double-nucleotide variants (Alexandrov et al., 2020). A similar analysis strategy has also been widely applied to somatic mutations identified from healthy human tissues or cells (Lodato et al., 2018; Martincorena et al., 2018) as well as from cultured cell lines (Kucab et al., 2019).

**FIGURE 1:**
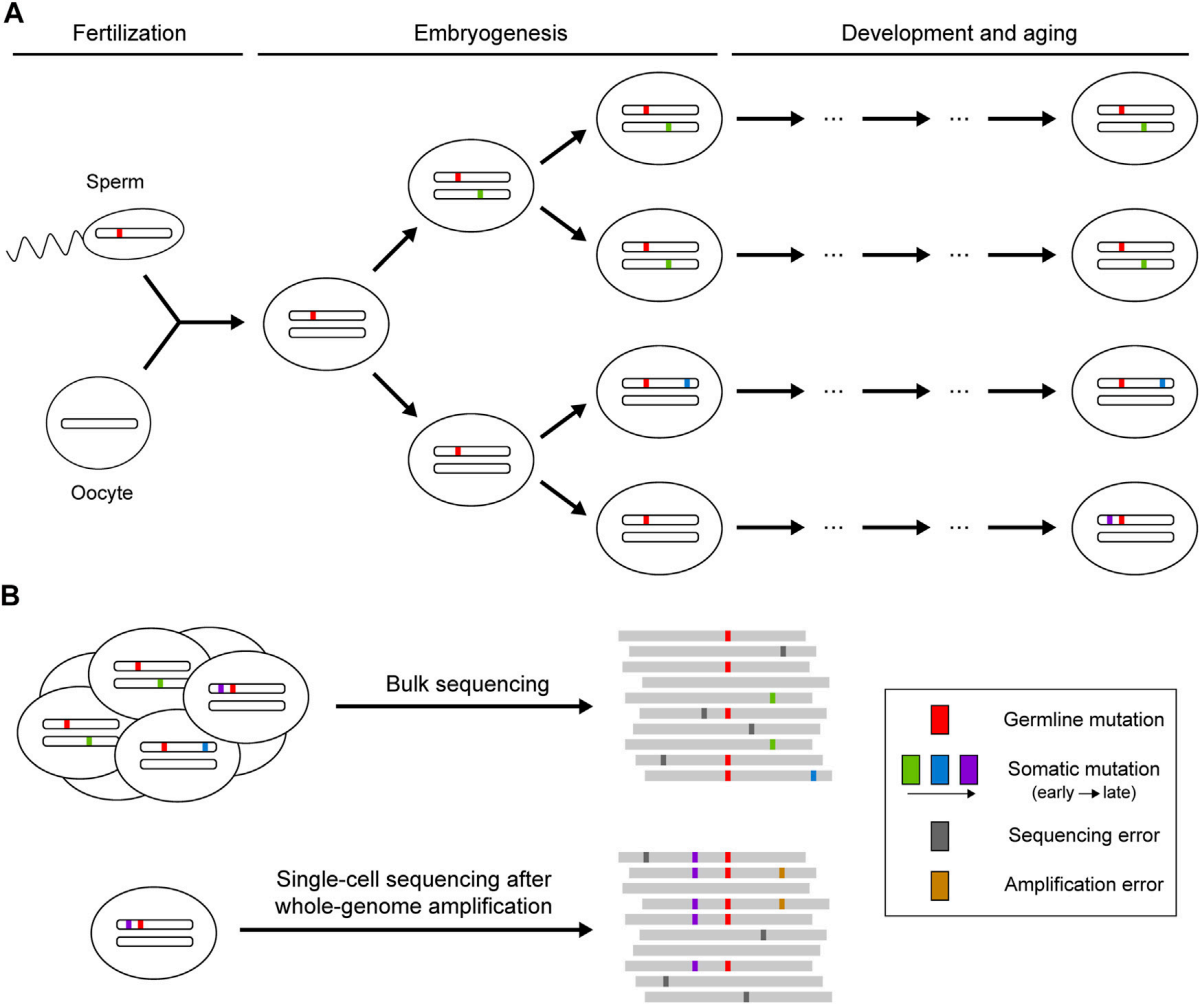
Occurrence of somatic mutations and their identification in next-generation sequencing data. **(A)** Somatic mutations that occur postzygotically after fertilization. Mutations arising during embryogenesis or under clonal expansion (green and blue) are shared in a fraction of the cell population, whereas mutations accumulating during the aging process (purple) may only be present in a single cell. **(B)** Identification of somatic mutations using bulk or single-cell sequencing. Bulk sequencing is suitable for detecting somatic mutations shared across multiple cells, though mutations with low allele fractions are difficult to distinguish from sequencing errors. Private somatic mutations can be detected with single-cell sequencing, but the whole-genome amplification before sequencing may introduce additional artifacts resulting from amplification errors.

Theoretically, sequencing reads from reference and mutant alleles of a given mutation should follow a binomial sampling process, where the expected number of mutant reads is positively correlated with total depth and mutant allele fraction. The mutant allele fraction is one of the key variables for somatic mutation detection, which is largely determined by the timing of the occurrence of the mutation and the selective pressure acting on the cell carrying the mutation (Figure 1). Somatic mutations occurring during embryogenesis or subjected to clonal expansion can achieve high allele fractions (>1%) in the cell population so that such mutations can be detected when sequencing bulk samples at high depth (Huang et al., 2018). However, next-generation sequencing (NGS) is not perfect: the error-prone processes of base-calling and alignment can produce ubiquitous technical artifacts that resemble true somatic mutations (Ma et al., 2019). Random variation and systemic bias in sequencing cause the deviation of allele fractions of heterozygous germline mutations from the expected 50%, which can also lead to false calls of somatic mutation. More recently, single-cell sequencing has been developed as a powerful strategy to enable identification of somatic mutations that are carried by a very small number of cells or that are even restricted to a single cell (Baslan and Hicks, 2017). Due to the low DNA content in every single cell, various methods have been applied to amplify genomic DNA before sequencing (Gundry et al., 2012; Chen et al., 2017; Gonzalez-Pena et al., 2021), but they also introduce numerous amplification errors and severe coverage unevenness that need to be addressed for somatic mutation calling.

### Calling Somatic Mutations From Bulk DNA Sequencing Data

Early attempts on somatic mutation calling were made in cancer studies, where the sequencing data from a tumor sample were typically compared to a matched normal control sample obtained from the same donor. Strelka (Saunders et al., 2012) and VarScan2 (Koboldt et al., 2012) compared mutant allele fractions between tumor and normal samples to test whether any given site showed a significantly higher fraction in the tumor sample. JointSNVMix (Roth et al., 2012) further considered the base-quality information and deployed a Bayesian model to jointly analyze tumor and normal samples, in which germline mutations could be ruled out if they were predicted to be present in both samples. Moreover, MuTect (Cibulskis et al., 2013) generated a probabilistic model to calculate the likelihood of the presence of a mutant allele that could not be explained by base-calling error or sample contamination, and then utilized a panel of normal samples to reduce false positives and filter out germline mutations. In addition to these statistical models, these somatic mutation callers also incorporated a series of error filters to further remove technical artifacts based on aberrant read alignment patterns, such as strand bias or poor mapping scores.

Although clonal expansion events led by driver mutations are not rare in healthy tissues, they usually involve relatively small clones, making it hard to attain high allele fractions in bulk tissue sequencing (Martincorena and Campbell, 2015). Moreover, the lack of matched control samples in non-cancer studies poses further challenges to somatic mutation identification in healthy individuals. MosaicHunter (Huang et al., 2014) addressed these difficulties by introducing a mosaic genotype into the Bayesian model to identify sSNVs without the need for control samples; it also designed more stringent empirical filters to achieve high precision when the signal-to-noise ratio is lower in non-cancer tissues. For whole-exome sequencing data, the additional exome enrichment steps in library preparation could result in over-dispersed distribution of mutant allele fractions when compared to binomial expectation (Huang et al., 2017); MosaicHunter and EM-mosaic (Hsieh et al., 2020) introduced beta-binomial models to capture the over-dispersion estimated from each whole-exome sample. MosaicForecast (Dou et al., 2020) leveraged machine-learning methods to incorporate multiple classifiers to distinguish somatic mutations from false positives, and demonstrated a better balance of sensitivity and specificity than previous methods where error filters had been empirically defined.

Targeted ultra-deep sequencing has been widely used as a cost-efficient strategy to increase sequencing depth and thus improve sensitivity in detecting somatic mutations, especially for screening mutations in cancer-related genes (Martincorena et al., 2015; Keogh et al., 2018). However, conventional somatic mutation callers designed for whole-genome or whole-exome sequencing usually cannot produce high-confidence calls of somatic candidates with lower allele fractions (<1%), because a large number of technical artifacts can reach allele fractions of 0.1–1% in ultra-deep sequencing data (Fox et al., 2014). To address this, RareVar (Hao et al., 2017) built a position-specific error model considering genomic contexts including mutation type and GC content, enabling identification of sSNVs with a 0.5% allele fraction. RePlow (Kim et al., 2019) utilized technical replicates of the same sequenced sample to estimate the background error rate during library preparation and sequencing, which greatly reduced false positives in ultra-deep sequencing data.

### Calling Somatic Mutations From Single-Cell DNA Sequencing Data

Somatic mutation in single-cell data has emerged as a powerful endogenous marker to comprehend underlying mutational mechanisms across different cell types (Brazhnik et al., 2020), and to reconstruct developmental lineage during embryogenesis (Bizzotto et al., 2021). Theoretically, somatic and germline heterozygous mutations should appear similarly at the single-cell level, both following a binomial distribution for allele fraction with an expected probability of 0.5; therefore, a bulk sample from the same individual is usually necessary to facilitate distinguishing the two types of mutations. Current whole-genome amplification (WGA) methods in single-cell sequencing can result in widespread amplification errors arising during multiple rounds of PCR, highly variable read coverage across the genome, and severe allelic dropout events when one allele of a genomic locus completely failed to be captured and amplified (Gawad et al., 2016).

Early pioneering works have demonstrated success in applying bulk-sequencing-based methods to sSNV calling in single cells (Wang et al., 2014; Lodato et al., 2015), despite potentially high false positive rates with the lack of refined modeling of single-cell-sequencing-specific features. Monovar (Zafar et al., 2016) derived the conventional binomial model by considering global allelic dropout and amplification error rates for every single cell estimated by using heterozygous germline mutations. SCcaller (Dong et al., 2017) further applied a kernel smoothing method which enabled the estimation of local allelic dropout across different genomic loci, and achieved better performance. To eliminate false positives arising during amplification, LiRA (Bohrson et al., 2019) and Conbase (Hard et al., 2019) utilized the read phasing information between somatic mutation candidates and adjacent germline heterozygous mutations, where only true mutations but not artifacts would be completely linked to one of the two alleles of a germline heterozygous mutation. Moreover, SCAN-SNV (Luquette et al., 2019) estimated genome-wide allelic imbalance using germline heterozygous mutations and then checked whether a somatic candidate had a similar level of allelic fraction to local expectation.

Single cells may share some somatic mutations if those mutations occurred in their common ancestral cell (Woodworth et al., 2017). Compared to mutations that are present in only a single cell, shared mutations can be more reliably called and distinguished from random amplification errors if somatic mutation callers can jointly consider sequencing data from multiple single cells or bulk cell populations. Monovar and Conbase applied a similar set intersection strategy, in which somatic mutations from every single cell were called independently and then only mutations recurrently called in multiple cells were considered as true clonal events, although Conbase showed a much lower false positive rate due to its usage of read phasing information. With the consideration of single-cell-specific allelic dropout and amplification error rates, single-cell MosaicHunter (Huang et al., 2020) incorporated the genotype probability of single-cell and bulk sequencing data into a single Bayesian graphical model where bulk data was generated either from the actual bulk cell population or an *in silico* mixture of multiple single cells, and outperformed other tools on calling clonal mutations.

### Calling Somatic Mutations From Non-DNA Sequencing Data

Somatic mutations can also be called from other types of sequencing data beyond DNA sequencing data. RNA-MuTect identified exonic somatic mutations from bulk RNA-seq data by comparing mutation calls against DNA sequencing of a matched control sample (Yizhak et al., 2019). Somatic mutation candidates from RNA-seq data need to be distinguished from RNA editing sites and germline mutations with allelic expression bias There are successful attempts on calling somatic mutations from single-cell RNA-seq (Vu et al., 2019) and ATAC-seq (Bizzotto et al., 2021) data, but these analyses were limited to re-capture mutations that had been identified by other DNA-based methods. Mitochondrial DNA is known to have a higher mutation rate than the nuclear counterpart, likely due to the abundant mutagenic oxidative radicals and lack of DNA repair machinery (Schon et al., 2012). A recent study demonstrated the possibility of calling mitochondrial somatic mutations in single-cell RNA-seq and ATAC-seq data and using the mutations as lineage markers (Ludwig et al., 2019).

### Conclusion and Future Perspectives

Many bioinformatic methods have been developed to study somatic mutation in healthy and diseased human samples using bulk or single-cell sequencing (Table 1). In bulk-sequencing-based methods, the detectable allele fraction of somatic mutation is largely restricted by the intrinsic base-calling error rate of ∼0.01–0.1% in current sequencing technologies. Molecular barcoding has been suggested as a promising solution since it generates a consensus sequence from multiple sequencing reads derived from the same DNA fragment and dramatically reduces the base-calling error rate (Hiatt et al., 2013; Hoang et al., 2016; Abascal et al., 2021); however, the requirement of high sequencing depth and efficient tools for consensus sequence calling currently prevents its broad application. On the other hand, alternative experimental methods have recently emerged to bypass the WGA step in single-cell DNA sequencing, including cell culture of isolated single cells into clones (Bae et al., 2018) or organoids (Behjati et al., 2014; Nanki et al., 2020), micro-dissection of monoclonal cells from tissue sections (Martincorena et al., 2015; Li et al., 2020), and even direct sequencing without pre-amplification (Zahn et al., 2017).

**TABLE 1:** A selected list of tools for somatic mutation calling.

Tool	Reference	Sequencing type	Detectable mutation type	Optimized for non-cancer data	Built-in genotyper	Matched control required	Base-quality-aware in genotyper	Joint analysis of multiple samples
Strelka	Saunders et al. (2012)	bulk DNA	Shared	No	Yes	Yes	No	Yes, with matched control
VarScan2	Koboldt et al. (2012)	bulk DNA	Shared	No	Yes	Yes	No	Yes, with matched control
JointSNVMix	Roth et al. (2012)	bulk DNA	Shared	No	Yes	Yes	Yes	Yes, with matched control
MuTect	Cibulskis et al. (2013)	bulk DNA	Shared	No^a^	Yes	Yes^a^	Yes	Yes, with matched control
MosaicHunter	Huang et al. (2014) Huang et al. (2017)	bulk DNA	Shared	Yes	Yes	No	Yes	Yes, with matched control or parents
SomVarIUS	Smith et al. (2016)	bulk DNA	Shared	No	Yes	No	Yes	No
EM-mosaic	Hsieh et al. (2020)	bulk DNA	Shared	Yes	Yes	No	No	No
MosaicForecast	Dou et al. (2020)	bulk DNA	Shared	Yes	No	No	NA	No
Shearwater	Gerstung et al. (2014)	bulk DNA, ultra-deep	Shared	No	Yes	Yes	No	No
RareVar	Hao et al. (2017)	bulk DNA, ultra-deep	Shared	No	Yes	No	No	No
RePlow	Kim et al. (2019)	bulk DNA, ultra-deep	Shared	Yes	Yes	No	Yes	No
Monovar	Zafar et al. (2016)	single-cell DNA	Shared and private	No	Yes	Yes	No	Yes, with other single cells
SCcaller	Dong et al. (2017)	single-cell DNA	Shared and private	Yes	Yes	Yes	Yes	No
LiRA	Bohrson et al. (2019)	single-cell DNA	Shared and private	Yes	No	Yes	NA	No
Conbase	Hard et al. (2019)	single-cell DNA	Shared	Yes	Yes	Yes	No	Yes, with other single cells
SCAN-SNV	Luquette et al. (2019)	single-cell DNA	Shared and private	Yes	No	Yes	NA	No
single-cell MosaicHunter	Huang et al. (2020)	single-cell DNA	Shared	Yes	Yes	No	Yes	Yes, with bulk or other single cells
RNA-MuTect	Yizhak et al. (2019)	bulk RNA	Shared	Yes	Yes	Yes	Yes	Yes, with matched DNA
SCmut	Vu et al. (2019)	single-cell RNA	Shared	No	No	Yes	NA	Yes, with matched DNA

a Later versions of MuTect, with dramatic improvement from the method described in the original paper, allow somatic mutation calling in non-cancer samples and without matched control.

In the past decade, genomic studies have benefited from the development of single-molecule sequencing technologies that can directly read nucleotide sequences from DNA or RNA molecules and deliver much longer reads than previously available NGS technologies (Logsdon et al., 2020). Long sequencing reads unlock the possibility of exploring repetitive genomic regions that are generally inaccessible with short-read sequencing and characterizing large and complex genetic variants involving copy number variation or structural variation (Ameur et al., 2019). New bioinformatic tools specialized for long-read sequencing have emerged for read alignment (Li, 2018) and variant calling (Sedlazeck et al., 2018) that have been successfully implemented in cancer studies (Nattestad et al., 2018; Aganezov et al., 2020). However, the relatively high cost of single-molecule sequencing limits its broad application to genome-wide detection of somatic mutations with low allele fractions since such detection requires high sequencing depth. Rapid advances in sequencing technologies and bioinformatic methods will allow more comprehensive identification and deeper understanding of somatic mutations in healthy and diseased human genomes in the future.
